# A Rare Presentation of Spontaneous Spinal Epidural Hematoma as Spinal Cord Compression and Complete Paraplegia: A Case Report and Review of the Literature

**DOI:** 10.7759/cureus.22199

**Published:** 2022-02-14

**Authors:** Nilesh Barwar, Nitish Kumar, Amit Sharma, Ajay Bharti, Raj Kumar

**Affiliations:** 1 Orthopaedics, All India Institute of Medical Sciences, Gorakhpur, Gorakhpur, IND; 2 Orthopaedics, All India Institute of Medical Sciences, Jodhpur, Jodhpur, IND

**Keywords:** spontaneous spinal epidural hematoma, thoracolumbar spine, paraplegia, operative intervention, laminectomy

## Abstract

Spontaneous spinal epidural hematoma (SSEH) is a serious but infrequent cause of profound neurological compromise of acute onset. It is often an atraumatic occurrence, and in around half of the cases, no etiology is identified. However, several causes such as arteriovenous malformation in the spine, use of anticoagulants in various cardiovascular diseases, and spinal trauma have been incriminated for its development. Here we encountered a case of SSEH following unregulated use of anticoagulants after a mitral valve replacement surgery. The patient had complete paraplegia with bowel and bladder involvement. The case was treated with decompressive laminectomy with regularization of her coagulation profile. Although she presented late to the healthcare center for the treatment, she showed a remarkable neurological improvement with gaining power worth near independent ambulation after one year of follow-up.

## Introduction

Spontaneous spinal epidural hematoma (SSEH) is a relatively rare condition, yet it is a sudden and profound atraumatic cause of neurological compromise. It is accounted for less than 1% of all spinal canal space-occupying lesions [1, 2]. SSEH is defined as bleeding within the epidural space without known traumatic or iatrogenic cause and has an estimated incidence of 0.1 in 100,000 per year [2-5]. The usual clinical presentation of SSEH is sudden pain in the back that may progress to partial motor deficit in the extremities to a complete neurological compromise, depending on the level and size of the lesion [6].

As it is a rapidly progressive space-occupying mass in the epidural space, it can produce devastating neurologic deficits within a short time frame of hours or days [7, 8]. Therefore, early diagnosis and appropriate interventions must be made diligently when a patient has symptoms concerned with SSEH. It can be idiopathic in many cases. However, certain causes like coagulopathy, vascular anomalies, malignancy, infections, and minor vertebral traumas are cited as potential causes [6]. In the present case, a young lady presented to our ED with sudden onset of paraplegia with bladder and bowel involvement following an episode of SSEH in the thoracolumbar region of her spine.

## Case presentation

A 22-year-old female presented to our orthopaedic department with complaints of backache and bilateral lower limbs sensory and motor deficit since last five days. She was a known case of rheumatic heart disease and had undergone mitral valve replacement surgery nine years ago. She was on oral vitamin K antagonist anticoagulant (acenocoumarol) after the surgery.

On neurological examination of bilateral upper limbs, sensory and motor functions were intact with normal deep tendon reflexes (DTRs). In the lower extremities, muscle tone was decreased with flaccidity. The motor power was assessed using Medical Research Council (MRC) grading, and it was zero (0/5) around the hips, knees, and ankles. DTRs were absent around the knees and ankles, the plantar reflexes were mute bilaterally, and the abdominal reflex was absent. There was complete involvement of the sacral segment of the spinal cord; perianal sensation, voluntary anal contraction, and sensation to deep anal pressure were absent. Sensory function was normal up to D11 dermatome, decreased from D12 to L2, and absent below L2 level. The bulbocavernosus reflex was absent.

Laboratory parameters such as complete blood counts, liver and kidney function tests, and erythrocyte sedimentation rate were within the normal limit. C-reactive protein was markedly elevated with anemia. The prothrombin time and International Normalized Ratio (INR) were marginally raised with normal activated thromboplastin time. On further inquiry, she admitted that before coming to the present hospital, she quit taking the anticoagulants in consultation with a physician at the nearby place after the appearance of weakness in the limbs. Further, it was found that her value of INR of one week before the admission was highly elevated (Table 1).

**Table 1:** Laboratory parameters. ESR: Erythrocyte sedimentation rate. Serial No. 12 shows marked elevation of the International Normalized Ratio.

Serial No.	Test	Patient’s value on admission	Reference range
1	ESR	08mm/1 Hour	0-20 mm/hour
2	High Sensitivity C-Reactive Protein	96.5 mg/L.	<1 mg/L
3	Hemogram	7.8 g/dl	12.0 to 15.5 g/dl
4	WBC	9.59 (10^3 /ul )	4.5-11 (10^3 /ul)
5	Neutrophils	68.5 (%)	55-70 (%)
6	Neutrophils	6.57 (10^3 /ul )	2.5-8.0 (10^3 /ul )
7	Lymphocytes	20.3 (%)	20-40 (%)
8	Platelets	291 (10^3 /ul )	200-500 ( 10^3 /ul )
9	Prothrombin Time (PT)	17.5Seconds	11 to 13.5 seconds
10	International Normalized Ratio	1.34 NA	0.8 to 1.1 NA
11	Activated Partial Thromboplastin Time (APTT)	36.9Seconds	30 to 40 seconds
12	International Normalized Ratio	6.00 NA (value one week before admission)	0.8 to 1.1 NA

**Figure 1:**
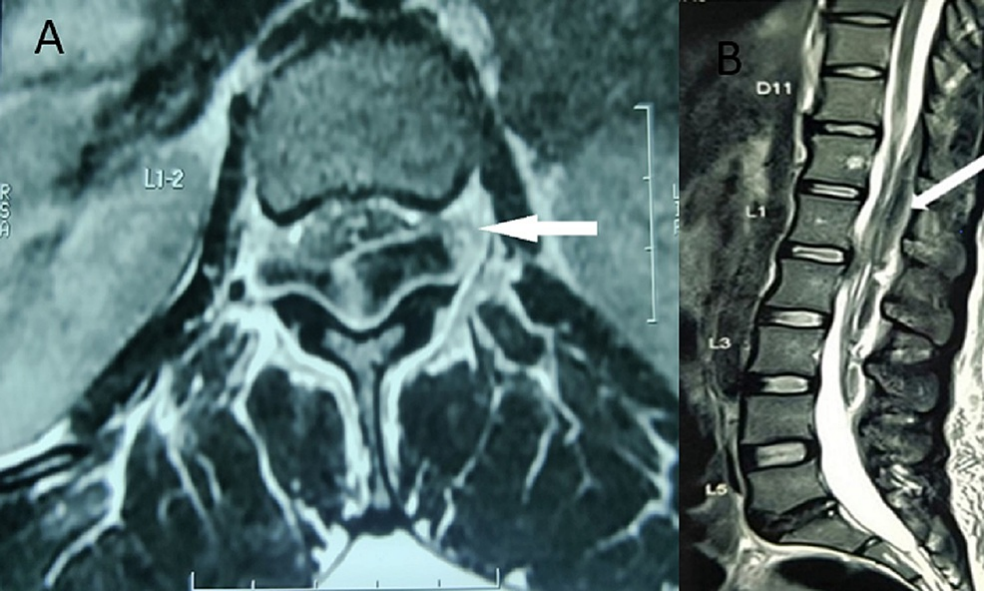
MRI axial (A) and sagittal section (B). T2-weighted images showing hypointense lesion at the thoracolumbar area. The mass is pushing the cord to the anterior side.

On radiological evaluation, the X-rays of the lumbosacral spine were unremarkable except for a mild lateral curvature to the right side with some thoracolumbar kyphosis. MRI of the whole spine revealed an elongated dorsal epidural mass in the thoracolumbar region of the spine extending from D11 to L4 level in epidural space, showing slightly high signal intensity on T1 and T1 fat-suppressed sequences and hypo-intense signals on T2-weighted images. A definite signal change in the spinal cord was not seen (Figure 1A-1B). Spinal angiography was conducted, but no vascular abnormality was observed.

Based on the clinical, laboratory, and radiological evaluation, she was diagnosed as a case of SSEH extending from D11 to L4 with complete paraplegia and bowel and bladder involvement. In addition, she had a picture of flaccid paralysis. Therefore, before taking her to the surgery, we consulted a hematologist to review her coagulation profile. 

Surgical management

**Figure 2:**
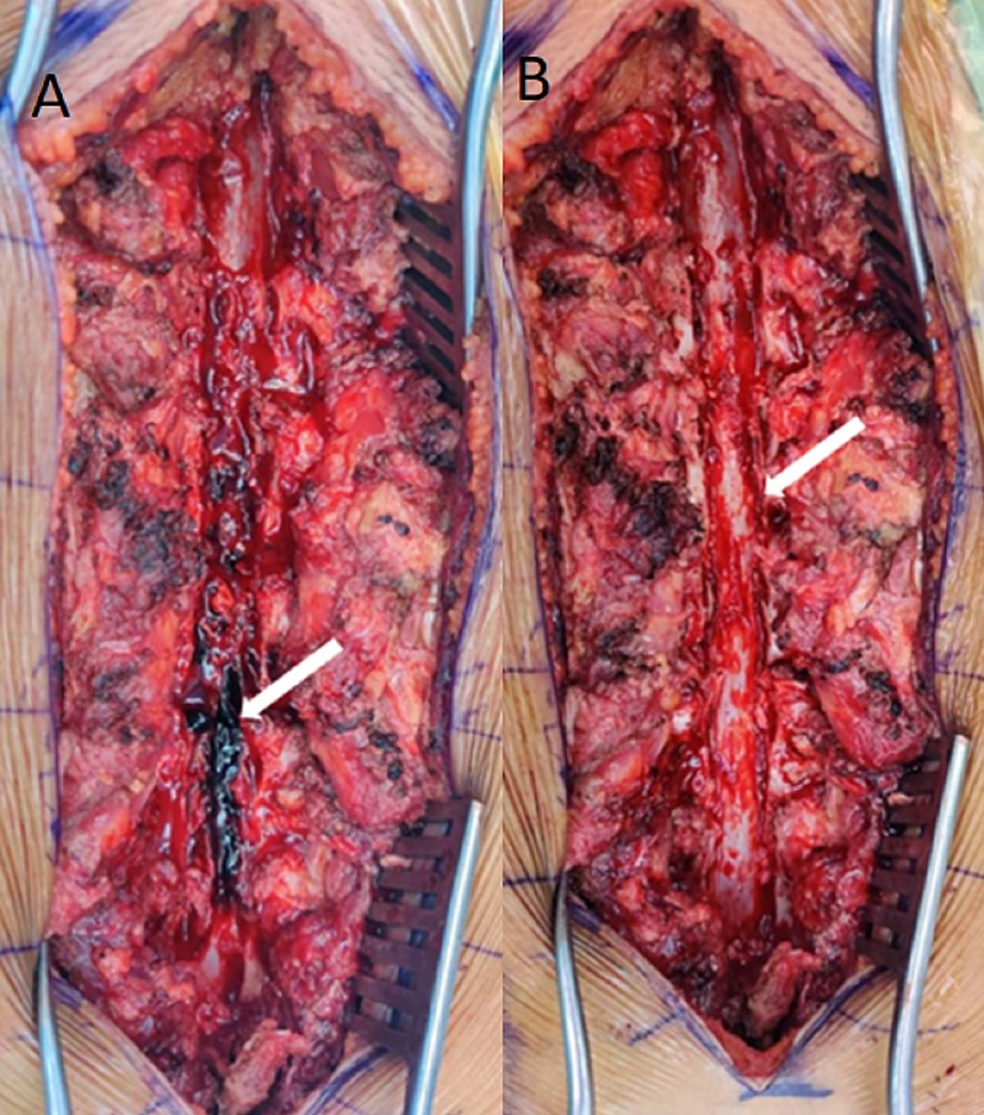
Intraoperative images of the surgical wound of the spine. 2A: Dark-colored clots of the hematoma. 2B: After laminectomy and hematoma clearance, the cord was decompressed.

Surgical decompression of the spinal cord was considered after due consultation with the hematology department. Laminectomy and posterior decompression were executed from D11 to L4 level under general anesthesia with the help of a BoneScalpel (Misonix, Inc., NY, USA). On opening the epidural space, extensive clots were found in the thoracolumbar region. Meticulous removal of the clots with penfields of appropriate size was done, and complete decompression of the spinal cord was achieved (Figure 2A-2B).

Post-operative management

Supportive care was provided in the post-surgical period. A hematologist reviewed the patient for regulating her coagulation profile. The doses of the anticoagulant "acenocoumarol" were reduced and adjusted. The INR test was done on a daily basis and was ensured in the range of 1.5 to 2.5 NA. She was discharged to home. The patient was counseled to get the INR report every week and advised to keep that in the prescribed bracket in consultation with a local physician. She was also counseled for taking care of bedsore prevention by doing respiratory and other exercises to prevent complications of recumbency.

Follow-up

The patient was closely followed up every month after suture removal. A significant improvement in the neurological function was observed at every visit. At the last outpatient department (OPD) visit at one-year post-surgery, she gained complete bladder and bowel control with normalization of her sensory function. In addition, the muscle power in lower limbs improved significantly.

**Figure 3:**
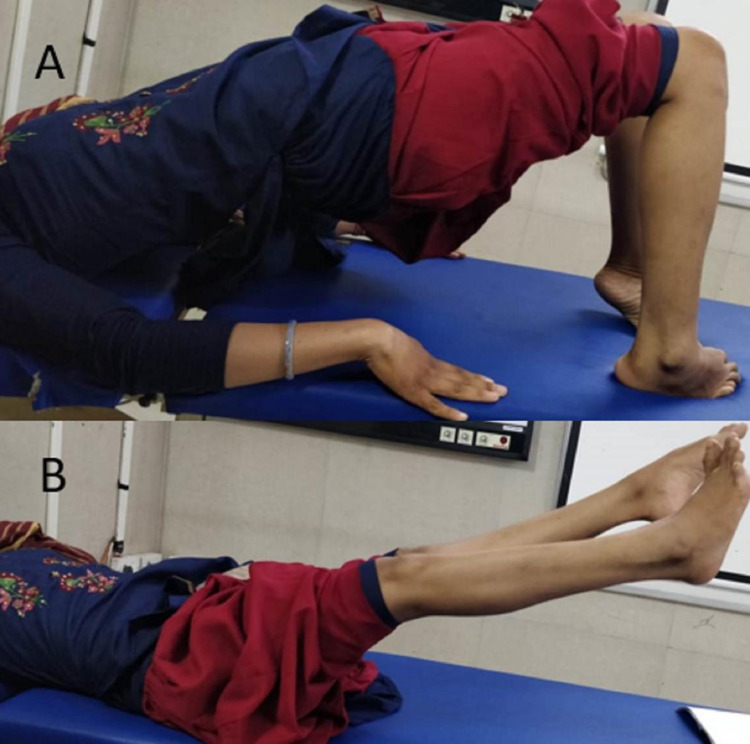
Patient photographs (one-year post-surgery). The motor power around hips, ankle (Figure A), and knees (Figure B) improved to the extent of ambulation.

Motor functions at one-year post-surgery: Hips 4/5, knees flexion 3/5, knees extension 4/5, flexor hallucis longus (FHL) 2/5, extensor hallucis longus (EHL) 3/5 (right side), EHL 1/5, and FHL 0/5 (left side). The power in plantar flexion improved to 4/5 on both sides (Figure 3A-3B), yet the ankle dorsiflexion was weak (2/5 on each side). The Babinski sign was positive bilaterally. There was an element of spasticity in the lower limbs. At the end of one year, she was walking with the help of a stick. Passive stretching was advised, and "ankle-foot orthoses" were given to deal with ankle dorsiflexion weakness.

**Figure 4:**
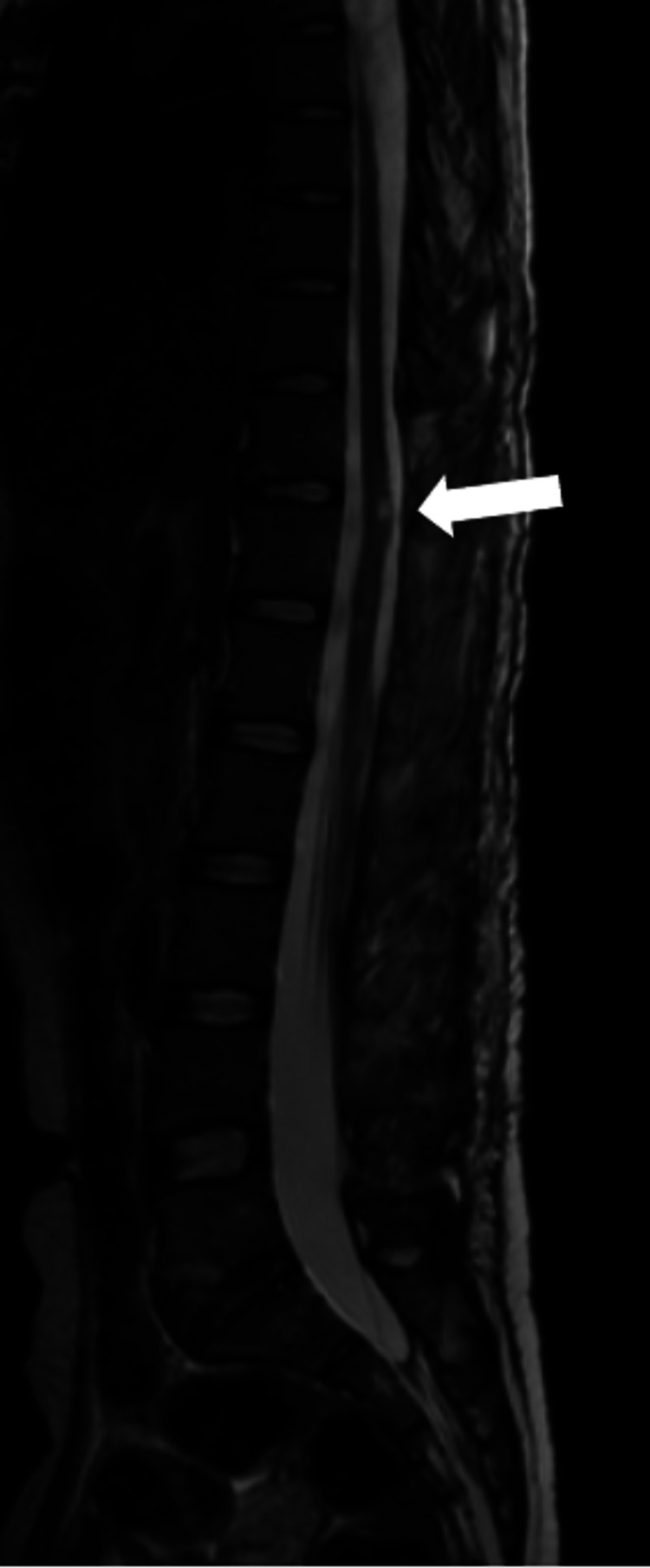
MRI sagittal section T2-weighted image (one year post-surgery). The MRI shows a completely decompressed cord with residual hyperintensity.

An MRI of her spine at one year revealed no residual compression of the neural structures. However, there were areas of hyperintensities in the cord at the level D11 to L1, suggesting a sequel of cord compression (Figure 4).

## Discussion

SSEH is a rare but significant neurological condition. Its incidence is most frequent after the fourth or fifth decade [9]. Men have more commonly been affected with the male-female ratio of 1.4:1 [10, 11]. Some predisposing factors such as anticoagulant therapy for prosthetic cardiac valves, therapeutic thrombolysis for acute myocardial infarction, hemophilia B, factor XI deficiency, long-term aspirin use as platelet aggregation inhibitors, and vascular malformation are suggested as potential causes of SSEH [12, 13].

In approximately 40% of cases, no exact etiology is found [10]. Areas of the spine that have high stress owing to being a junction of the relatively rigid and mobile spine, such as the cervicothoracic and thoracolumbar region are more commonly affected [9, 14]. There is an ongoing debate regarding the origin of the hematoma. Some authors have claimed that it arises from the epidural venous plexus as it does not have valves and the walls are thin and delicate. A sudden increase in pressure in the thoracic and abdominal cavity can directly be transmitted to the plexus. Activities such as sneezing, coughing, defecation, lifting heavyweight, etc., might be responsible for epidural bleeding [9, 10]. In contrast, other researchers proposed the spinal epidural arteries as the source of hemorrhage [13]. As the arterial pressure, unlike the venous pressure, is higher than the intra-dural pressure, the arterial cause of the hematoma formation seems to be the more likely cause [15].

Pain in the back, which might radiate to one or more limbs, depends on the amount and location of the hematoma. In addition, the clinical picture may confuse the attending physician as similar symptoms may arise from other clinical entities such as cerebrovascular accidents, myocardial infarction, meningitis, etc. [16, 17].

In most cases, the deficit is acute, with its onset ranging from hours to days. The quantum of neurological compromise varies in individuals, and it may range from a partial deficit to complete loss. For complete deficit, the scientific community is divided in its opinion as far as the treatment modality is concerned. Some authors have proposed an urgent surgical modality (within 36 hours) as the treatment of choice. The results of surgical decompression have been reported promising, even when the deficit is complete [18]. However, some authors advised conservative management even in the situation of complete deficit [10, 19].

In our case, as the coagulation profile was reasonably in the normal range, we could do the surgical procedure within 48 hours of the admission. After surgical decompression, neurology improved gradually, and bladder and bowel function recovered completely. Just as in spinal cord injury, the amount of neurological recovery depends on the severity of the neurological compromise before the operative intervention, and it is the most crucial prognostic tool [4, 5]. As the case was reported to us approximately one week after the onset of symptoms, the surgical decompression was delayed. Also, she had involvement of the cord along with the cauda equina. Hence, it seems the lower motor neuron (LMN) type injury resolved profoundly except for the ankle dorsiflexion. Because of the involvement of the cord at the thoracolumbar junction, the patient has a component of residual spasticity in the lower extremities. Despite the residual weakness, the patient is still able to ambulate with a stick.

## Conclusions

Whenever a case having an atraumatic neurological deficit of sudden onset is seen, one should have a vigil on the coagulation profile. A thorough history can reveal the clinical diagnosis of SSEH. For cases with profound or progressive neurological compromise, early surgical intervention in the form of decompressive surgery can be hugely rewarding. In addition, patients on anticoagulant therapy should be counseled to monitor their coagulation profile at regular intervals. Any unacceptable derangement can lead to catastrophic consequences.
